# Association between mandibular second molars calcification stages in the panoramic images and cervical vertebral maturity in the lateral cephalometric images

**DOI:** 10.4317/jced.56402

**Published:** 2020-02-01

**Authors:** Mohammad-Hossein Toodehzaeim, Elahe Rafiei, Seyyed-Hadi Hosseini, Alireza Haerian, Milad Hazeri-Baqdad-Abad

**Affiliations:** 1 Associate Professor, Department of Orthodontics, Faculty of Dentistry, Shahid Sadoughi University of Medical Sciences, Yazd, Iran; 2 Assistant Professor, Department of Orthodontics, School of Dentistry, Shahid Sadoughi University of Medical Sciences, Yazd, Iran; 3 Dental Student, School of Dentistry, Shahid Sadoughi University of Medical Sciences, Yazd, Iran; 4 Assistant, Department of Orthodontics Dentistry, Dental School, Shahid Sadoughi University of Medical Sciences, Yazd, Iran

## Abstract

**Background:**

Determination of maturation and evaluation of growth potential is extremely important in clinical orthodontics. The purpose of this study was to evaluate the diagnostic performance of the mandibular second molar calcification stages for identification of growth phases.

**Material and Methods:**

In this cross-sectional descriptive study, samples were derived from panoramic radiographs and lateral cephalograms of 125 subjects (61 males and 64 females) with age ranging from 8 to 17 years and estimates of dental maturity (Demirjian Index [DI]) and skeletal maturity (Cervical vertebral maturation indicators CVMI]) were made. Correlations between DI and CVMI were shown by Spearman’s correlation. The diagnostic performance of the DI for the identification of the growth phase were evaluated using positive likelihood ratios (LHRs), with a threshold of ≥10 for satisfactory performance.

**Results:**

Correlations between second molar calcification and growth phase were 0.819 for females and 0.805 for males (*P*-value< 0.0001). LHR ≥10 was only observed for the identification of the post-pubertal growth phase for the H stage of the second molar.

**Conclusions:**

In spite of the high correlation coefficients between mandibular second molar calcification stages and skeletal maturity, these stages are reliable only for the identification of the post pubertal growth phase.

** Key words:**Skeletal maturation, demirjian Index, mandibular second molar.

## Introduction

Determining the stage of development and examining growth, plays an important role in clinical orthodontics in recognizing the appropriate and effective time period for treatment of various malocclusions (1,2).

The chronological age isn’t very precise in determining the stage of development in different children and is not always reliable and this leads to the development of the biologic age concept (3). Biologic age can be estimated in accordance to morphologic age (based on height), dental age, sexual age and skeletal age. Corresponding relationships between skeletal, body and sexual maturity have been identified in numerous studies (4,5).

Skeletal maturity is a reflection of the individual’s physiologic development (6). The best index for examining skeletal development is an x-ray of the wrist. However, the changes in size and shape of cervical vertebrae observed in a lateral cephalometric radiograph in growing individuals has been considered as an index for determining the stage of development to prevent additional exposures for wrist radiographs (5). The efficiency of predicting growth spurt by examining cervical vertebrae (instead of wrist radiographs) has been displayed in the Hassel and Farman or Baccetti *et al.* methods (7).

Analysing dental development based on radiographs is closely correlated to skeletal development (8) The stage of dental development can be determined by analysing the stage of tooth eruption; however, examining the tooth formation stage has been recommended as a more suiTable method for determining the stage of development (6).

Numerous studies have pointed out a relationship between the calcification phase of the upper second molar and maturation of cervical vertebrae (3,9,10).

Even though many studies have reported a correlation between the calcification process of the second mandibular molar and the growth and development process, few studies are available that investigated the diagnostic function of this correlation. The aim of this study is to investigate the diagnostic ability of using the calcification process of the mandibular second molar to identify the growth stage in a population of Iranian boys and girls.

## Material and Methods

This was a comprehensive-analytic cross-sectional study carried out on 125 panoramic radiographs and lateral cephalometric radiographs (each gender made up for approximately half of the samples) which were obtained from patients who had visited the orthodontics department in Yazd school of dentistry. The patient files were from the year 2011 to 2015, such that 25 files were chosen randomly from each year.

The following were the criteria for entering the study:

1- Aged between 8-17 years old

2- No history of systemic disease and in complete health

3- The panoramic and lateral cephalometric radiographs meet good diagnostic quality and both be available in the patient’s file.

4- The second molar should not be missing

All of the radiographs were analysed on a negatoscope by a calibrated dental student and in order to check the coefficient of correlation an experienced orthodontist was consulted, such that 40 panoramic and lateral cephalometric radiographs were chosen randomly from all of the samples and analysed by an experienced orthodontist.

The skeletal development was analysed using the rectified cervical vertebrae maturation method (CVM) proposed by Baccetti (cs1-cs6) on a lateral cephalometric radiograph using the C2 to C4 cervical vertebrae (11).

Cervical stage 1 (cs1): The inferior borders of all three vertebrae (C2-C4) are flat. The body of the C3 and C4 vertebrae are trapezoid shape (the superior border of the vertebrae are tapered from the posterior to anterior). The growth spurt of the mandible usually occurs two years after this stage.

Cervical stage (cs2): The inferior border of the C2 vertebra is concave. The body of C3 and C4 is still trapezoid. The growth spurt of the mandible usually occurs 1 year after this stage.

Cervical stage 3 (cs3): There are concavities on the inferior border of C2 and C3. The body of C3 and C4 can be trapezoid or a horizontal rectangle. The growth spurt of the mandible occurs in this year after this stage.

Cervical stage 4 (cs4): The inferior border of C2, C3 and C4 is concave. The body of C3 and C4 is shaped as a horizontal rectangle. The growth spurt of the mandible occurs between 1 to 2 years before this.

Cervical stage 5 (cs5): The inferior border of C2, C3 and C4 is concave. At least one of the C3 or C4 vertebrae are square shaped. The body of other cervical vertebrae are a horizontal rectangle if not square shaped. The growth spurt of the mandible occurs 1 year before this.

Cervical stage 6 (cs6): The inferior border of C2, C3 and C4 is still concave. The body of at least one of the C3 or C4 vertebrae is a vertical rectangle. The body of the rest of the other cervical vertebrae are square if not a vertical rectangle. The mandibular growth spurt has ended two years before this stage.

Different growth stages are divided as follows:

1) CS1 and CS2 are pre pubertal growth stages.

2) CS3 and CS4 are pubertal growth stages.

3) CS5 and CS6 are post pubertal growth stages.

The calcification stages of the left second mandibular molar were analysed using panoramic radiographs based on the Demirjian method.

The eight stages of dental mineralisation according to Demirjian method are as follows (12):

Stage A: Initial crown calcification, different calcifications are not attached to each other

Stage B: Different calcification are attached to each other

Stage C: Enamel formation is complete in the occlusal area and dentin formation has begun. The pulp chamber is lunar shaped and pulp horns are not visible.

Stage D: Crown is formed up to the CEJ. Root formation and differentiation of pulp horns has begun.

Stage E: Root length is shorter than crown length. Pulp horns are more developed than the previous stage. Calcification of the root furcation in molars has begun.

Stage F: Root length is equal to or greater than crown length. The furcation in molars has developed to the stage where the root shape is differentiable.

Stage G: Apex of the root is relatively open. In molars, only the distal root is open.

Stage H: The root apex is completely closed (distal root in molars).

After data collection it was evaluated using SPSS version 17 software. Statistical analysis was carried out using Spearman correlation test. To calculate the diagnostic ability of each stage of calcification of the second molar in identifying the correct stage of development, the positive likelihood ratio(sensitivity/1-specificity) with a confidence interval of 95% was used. A positive likelihood ratio of 10≤ was considered as a significance level of diagnostic ability.

## Results

The Kappa coefficient for the second molar and CVM between the student and orthodontist was 0.97 and 0.99 respectively which was high and accepTable value.

 Table 1 shows the frequency of cervical vertebrae maturity according to chronologic age in each age group. As can be observed in each age groups, females were in a higher CVM stage, except for the first and second age group. It is shown that in the last age group (16-17 years), most males are in CVM stage 5 (63.3%) whilst most female are in CV stage 6 (64.3%).

**Table 1:**
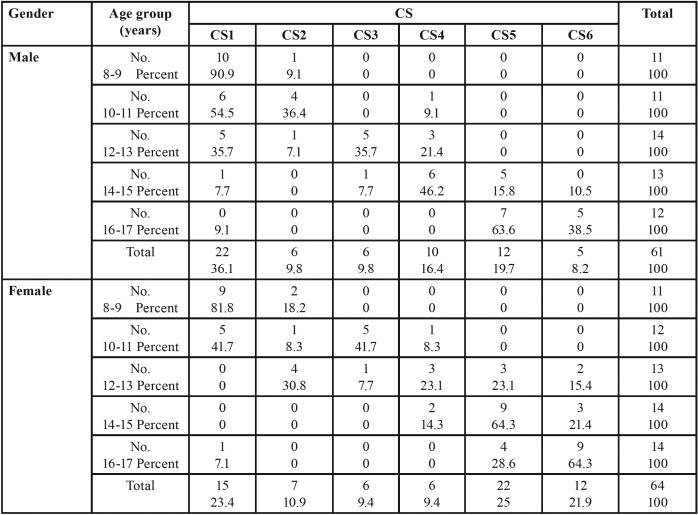
Frequency distribution of skeletal puberty stages according to chronological age.

As shown in  Table 2, most of the second molars before the growth spurt were in stage E. During the growth spurt the maturity of most of the second molars lies in stage G for males and stage F for females. After the growth spurt has ended the apex of the majority of teeth is closed (stage H) whereas in females the majority of the apexes are open (stage G).

**Table 2:**
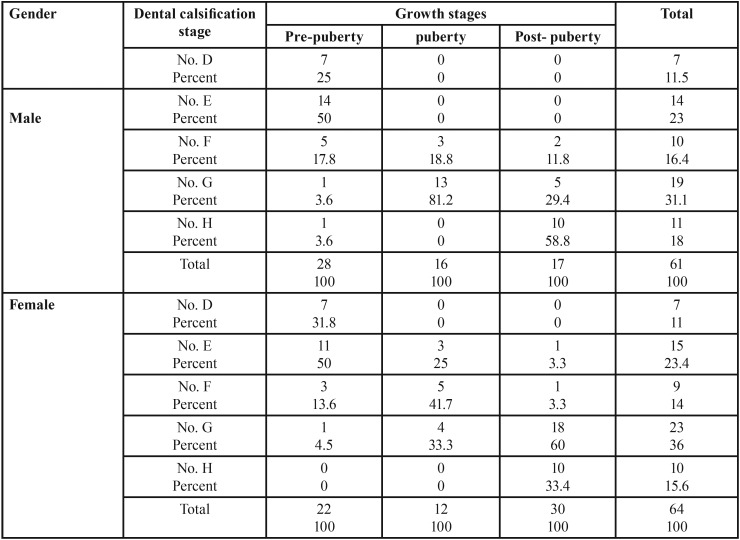
Frequency distribution of second molar calcification stages according to growth stages.

The Spearman correlation coefficient between the second molar calcification stage and maturity stage in males is r=0.805 and r=0.908 in females with a *p* value of *p*<0.0001 which represents a positive and significant correlation.

 Table 3 shows the positive likelihood for each stage of calcification of the second molar. A LHR value of ≥10 can only be seen in stage H (32/76) in identifying the growth period after the pubertal growth spurt. Also the likelihood ratio of the calcification stage E is 9.43 for the pre-pubertal growth stage, which is relatively high but still below 10.

**Table 3:**
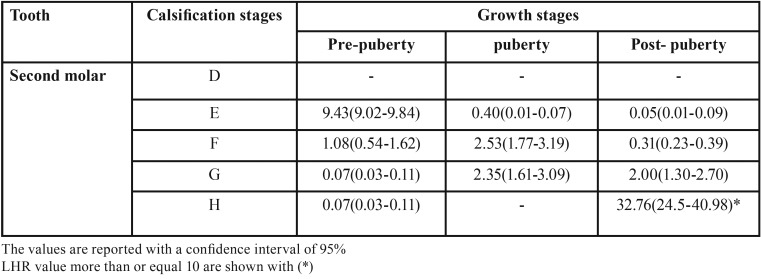
Positive likelihood ratio for each of the calcification stages of the mandibular second molar in determining the growth stages.

## Discussion

Many researchers have observed during their studies that the best time for functional orthodontic treatments is the pubertal growth spurt period (6,13). The growth spurt can be analysed based on indices such as height, skeletal maturity of the wrist and maturity of cervical vertebrae (14-19).

Considering that these racial differences affect dental and skeletal maturity (9) and few studies exist in this field on the Iranian population, carrying out a study to investigate this population can be useful.

Using a panoramic radiograph to evaluate skeletal maturity by stages of mandibular second molar is preferred over the traditional hand-wrist technique. Panoramic radiographs are a routine diagnostic radiograph, therefor patient radiation is limited (3).

In this research Demirjian method was used to examine the dental development stages. In this method the shape of the crown and the ratio of root length to crown length is used and no digit measurement is used to rule out the possibility of foreshortening and elongation caused by radiographic errors. Also in this method the amount of tooth formed is considered instead of the eruption stages of the tooth. The eruption phases of the tooth are affected by environmental factors and dietary habits, systemic disease and its validity is questionable (20).

In this study, instead of eruption, we used calcification stages as the indicator for dental maturation, which is regarded as more reliable in studies (3).

The apex of the second molar forms completely around 16-years, which is the latest among other mandibular teeth except the third molar. Therefore, this tooth was chosen to analyse the skeletal maturity. Although the third molar’s apex closes during a longer period of time (closure at the age of 22 years) due to a relatively high chance of third molar missing it was not used in this study (21). The maxillary second molar is not used due to possible super impositions caused by anatomic structures. The present study was taken up to evaluate the reliableness of utilizing the formative phases of mandibular second molar as a marker for development. This tooth offers predilection over other teeth since its advancement will in general proceed over a more drawn out period and until a later old age.

The modified Cervical Vertebrae Maturity method (CVM) proposed by Baccetti (cs1-cs6) was used in this study to analyse the cervical vertebrae maturity on lateral cephalometric radiographs using the C2 to C4 cervical vertebrae. Compared to the wrist radiograph method, this approach is more reliable and eliminates the necessity for exposing the wrist to additional x-rays (11). This was also reported in Imani Moghaddam *et al.* (22), Pancherz *et al.* (23) and Grave *et al.*’s (24) studies. Only one stage of skeletal maturity is observed in each lateral cephalometric radiograph. In this study it was shown that for each similar growth stage, boys have a larger frequency distribution in one stage of advanced dental development. This finding is similar to the studies carried out by Krailassiri (25), Uysal (10), Chertkow (26) and Basaran (1). They showed that tooth calcification compared to wrist and hand maturity follows a more advanced pattern in boys. In other words, in the same maturity stage of cervical vertebrae, boys present a higher distribution of frequency in one stage of advanced dental maturity.

In this study a statistically significant correlation was observed between the calcification stages of the second molar and the stages of skeletal maturity which is similar to the results of the studies carried out by Basaran (1), Chen (6), Cossellu (9), Uysal (10) and Kumar (3). In a study carried out by Valizadeh *et al.* same result was reported in a population of Iranian girls.

Kumar *et al.* showed that in CS3 and CS4 almost all second molars in both males and females are in stages G and F and suggested that the stages G and F, signify pubertal growth spurt (3). Some studies have also indicated that the G (25) and F (26) calcification stages of the mandibular canine have a high correlation with the pubertal growth spurt. Nevertheless, none of these studies calculated the diagnostic performance of dental maturity phases in analysing the pubertal growth stages and their reports were based on the analysis of distribution of frequency for dental maturity based on skeletal maturity stages.

Positive likelihood ratio (LHR) is the most important characteristic for a diagnostic test (calcification phases for teeth) that can be used to determine the presence or lack of a disease (skeletal maturity stages) in an individual.

The more the result of a positive likelihood ratio for a test is bigger than 1, the higher the probability of the disease (for example any of the skeletal maturity stages) for the individuals with positive test results (for example any of the dental calcification stages). Usually tests with a positive likelihood bigger than 10, are more suiTable for clinical application. The positive likelihood ratio considers both sensitivity and specificity (27-31).

Perinetti *et al.* calculated LHR and reported that stages D and E for the second mandibular molar are more reliable indexes to identify the pre-pubertal growth stage (32). In the current study, even though stage E has a high diagnostic ability for pre-pubertal growth stages, but this diagnostic ability is not statistically significant.

In another study by Perinetti *et al.*, the LHR was calculated and the H stage for the mandibular second molar was reported to be a more reliable index for identifying the post-pubertal growth stage (33) which correlates with the results of the current study.

A limitation of this study is the fact that, the pre-pubertal and post-pubertal growth stages are the only stages that the mandibular second molar calcification may be helpful. So, exact information about the time of growth spurt cannot be obtained by dental calcification. Another limitation is the sample size which, can affect the result and increasing it may provide more reliable outcomes.

## Conclusions

Even though there is a high correlation between the calcification stages of the mandibular second molar and the growth stages, the calcification stages of this tooth only possess diagnostic performance for post-pubertal growth stages. Therefore, using the calcification stages of the mandibular second molar to estimate the appropriate time for treatments to be carried out during the post-pubertal growth phase can be a useful index however, it is not recommended for treatments that need to carried out during pubertal and pre-pubertal growth stages.
